# Introducing onsite antenatal syphilis screening in Burkina Faso: implementation and evaluation of a feasibility intervention tailored to a local context

**DOI:** 10.1186/s12913-017-2325-x

**Published:** 2017-05-30

**Authors:** Fadima Yaya Bocoum, Grissoum Tarnagda, Fabrice Bationo, Justin R. Savadogo, Sarata Nacro, Séni Kouanda, Christina Zarowsky

**Affiliations:** 1 Département biomédical et santé publique, Institut de Recherche en Science de la Santé (IRSS), 03 BP: 7192 Ouagadougou 03, Burkina Faso; 2 University of Western Cape, School of Public Health, Cape Town, South Africa; 3 Ministère de la santé, district sanitaire de Kaya, Kaya, Burkina Faso; 4 Ministère de la santé, laboratoire du centre hospitalier régional de Kaya, Kaya, Burkina Faso; 5 Institut Africain de Santé Publique, Ouagadougou, Burkina Faso; 6 CR-CHUM, ÉSPUM, Université de Montréal, Montréal, Canada

**Keywords:** Syphilis, Screening, Antenatal care, Feasibility, Point of care test, Burkina Faso

## Abstract

**Background:**

Although the advantages of introducing point of care testing for syphilis in antenatal care (ANC) are well documented, there is little evidence on how to address structural issues within health systems. A better understanding of how these interventions work in a range of settings and contexts is needed in order to overcome bottlenecks at health system level. To better understand the relationships between implementation and context we developed and implemented an intervention focused on integrating a rapid screening test for syphilis in ANC services in rural primary health care facilities in Burkina Faso. This manuscript describes the intervention and reports on feasibility and acceptability of the intervention, the facilitators and barriers to the implementation of this intervention and the likelihood that point of care test for syphilis will become routinely incorporated in practice.

**Methods:**

In Kaya Health and Demographic Surveillance System (Kaya HDSS), all 7 primary healthcare facilities were selected for intervention in 2013. A participatory approach was used to design and implement an antenatal syphilis screening intervention. The Normalization Process Model (NPM) proposed by May et al. was adapted in order to identify barriers and facilitators and to explore the likelihood to become routinely incorporated in practice. Registers, Observations (*n* = 14 ANC 1) of interactions between patients and health workers during ANC and interviews with health workers (*n* = 14) were our data sources.

**Results:**

An intervention that included onsite training, provision of supplies and medicines, quality control and supervision was implemented in 7 health facilities in 2013. Rapid syphilis test and treatment were delivered during ANC within the examination room with no specific additional mechanism regarding staff organization. The perceived barriers were lack of training of all staff, workload, stock-outs of consumables and lack of motivation of staff. Key facilitators included political environment, ease of use of test and acceptability to pregnant women.

**Conclusions:**

Onsite testing for antenatal syphilis is a feasible and acceptable intervention in ANC at primary health facility in Burkina Faso. The point-of care test for syphilis is more likely to be acceptable by health workers as routine service and incorporated as a normal practice.

**Trial registration:**

The study was retrospectively registered on ClinicalTrials.gov under the Trial Registration Number NCT03156751.

## Background

Syphilis in pregnancy poses major health risks for the mother and the fetus [1] and also increases the risk of HIV transmission [2]. The World Health Organization (WHO) estimates that two million pregnant women each year are infected with syphilis globally [3]. The risk of vertical transmission could be up to 80% in early latent syphilis [3]. Approximately 1.2 million pregnant women with syphilis transmit the infection to their newborn every year [4]. Each year, maternal syphilis is responsible for at least 50,000 spontaneous abortions or stillbirths and 500,000 premature births of babies infected with congenital syphilis or who have low birth weight [3]. In Burkina Faso, prevalence in pregnant women was 1.9%, with notable regional variations [5]. In 2011 in Kaya a semi urban setting the prevalence was 4.3% whereas in Ouagadougou the capital city it was 2.1% [5].

Maternal syphilis is detectable by serological screening and is entirely treatable with penicillin. Consequently, screening and treatment for syphilis has been recommended as a routine part of antenatal care in many countries [6, 7]. Unfortunately, antenatal syphilis screening is often poorly implemented in many sub-Saharan African countries [8]. The influence of health systems issues on prenatal syphilis screening has been documented in several countries, such as Bolivia, Kenya and South Africa [9]. Yaya Bocoum et al. have documented constraints related to syphilis screening at primary health care facilities (CSPS) in Burkina Faso [10].

Syphilis screening in primary health care facilities during antenatal care (ANC) in Burkina Faso is conducted using a syndromic approach, but the majority of syphilis cases are asymptomatic. Testing is available at the hospital laboratories and conducted with a *venereal diseases research laboratory* (VDRL) test or with a *Treponema pallidum hemagglutination assay* (TPHA) which require qualified personnel, laboratory equipment, and a source of electricity, which limits their utility for primary health care facilities.

Currently, there are several available specific, rapid syphilis tests that are simple to use and could be implemented at primary health care settings [11]. These rapid tests for syphilis screening are available in Burkina Faso, and four of them have had their performance and operational characteristics assessed [12]. Unfortunately they are not widely adopted for ANC services.

Interventions to improve the outcomes of antenatal syphilis screening have been implemented in low and middle income countries where most congenital syphilis cases occur [13]. These interventions showed advantages of screening with rapid diagnostic tests and treatment of syphilis among pregnant women such as cost effectiveness [14–16]. Although the advantages of introducing point of care testing for syphilis in antenatal care are well documented, there is little evidence on how to address the structural issues within the health system [17]. A better understanding on how the interventions work in a range of settings and contexts is needed in order to overcome bottlenecks at health system level. When implementing any intervention, how – indeed whether – it works depends on elements of context such as socioeconomic background, health system structure and dynamics, and other factors [18]. It is with a view to better understanding the relationships between implementation and context that we developed and implemented an intervention focused on the integration of a rapid test for syphilis screening in the ANC services in primary health care facilities in Burkina Faso.

Revised UK Medical Research Council guidance on evaluating complex interventions [19] recommends describing the intervention and context and reporting on all stages of the process including feasibility evaluations.

The objectives of this study were to document operational implementation of a pilot integration of a rapid test for syphilis screening into routine antenatal services and analyze the experience in order to draw lessons for scaling up maternal syphilis screening in Burkina Faso. This manuscript reports on the experience of implementing antenatal syphilis screening with a point of care test, the identification of barriers and facilitators to integrating this intervention into routine ANC in rural Burkina Faso. Finally it reports on the likelihood that point of care test for syphilis will become routinely incorporated in practice.

### Study site – kaya HDSS

The intervention was nested in the Kaya Health and Demographic Surveillance System (Kaya HDSS), located in the Kaya health district in the North Central region of Burkina Faso, 100 Km from Ouagadougou, the capital city. It covers seven urban areas and 18 villages of the health district. By the end of 2011, 64 480 inhabitants living in 10 587 households were being followed. The population is very young as 55.5% are under 20 years of age. The Mossi are the predominant ethnic group and Islam is the main religion (78.9%). The majority of the population of the HDSS (53.3%) has not attended school. Subsistence farming and livestock breeding are the two main occupations. Artisanal mining is also significant. Within Kaya HDSS, dirt roads and bush paths provide means of access to the villages. These roads present a challenge for users especially during the rainy season when there are floods [20].

### Health facilities

Within Kaya HDSS, there are seven public primary health facilities that offer ANC, one faith-based health center and one regional hospital. The faith-based facility and the hospital do not offer ANC but their laboratories offer VDRL and TPHA tests for syphilis. In 2012, a pharmacy began offering a rapid test for syphilis but due to low demand they stopped selling the test.

All seven public primary health facilities, four urban and three rural, were selected for the intervention. All facilities have a maternity, dispensary and drug shop. There are 2 to 3 health workers staffing a rural facility compared to an average of 7 in urban facilities. Primary health care facilities are led by a state registered nurse and staffed by cadres including nurse, midwife, auxiliary midwife and outreach health workers. Nurses and midwives have formal training that includes syndromic STI case management.

The drugs for STI are standardized kits that are subsidized by the national committee for HIV and STI control. Every year kits are ordered by the national committee for HIV and STI control and supplied to the district for each facility. Frequently, drugs from these kits are expired or out of stock depending on the facility.

Syndromic STI case management and reference for laboratory test were used for syphilis screening in these facilities until the time of the intervention.

## Methods

### Design of the intervention

The main strategy of the intervention was the development and implementation of a decentralized model of syphilis screening among pregnant women. The components of this intervention included: (1) providing onsite training of health workers involved in ANC, (2) providing supplies and drugs to health facilities for diagnosis and treatment (3) implementing a quality control system, (4) and supervision and monitoring. Before the implementation a preparatory phase of formative research was undertaken.

### Preparatory phase

A situation analysis and evaluation of health worker and community perspectives on syphilis screening was the first stage of this project [10]. An evaluation of the performance of four available onsite rapid syphilis tests in Burkina Faso was also conducted in order to select the test. The results of this evaluation are reported elsewhere [21]. The selected point of care test for syphilis was Alere TM Determine TM Syphilis TP (AlereTM Medical Co Ltd, UK). Before the implementation, the head of the health district received information on the objectives and procedures of the intervention.

A workshop was organized with health workers from each of the selected facilities. Participants were staff who regularly performed antenatal care. During the workshop, the importance of decentralizing antenatal syphilis screening, case management of syphilis positive cases, including treatment, counseling and the components of the intervention and a manual were presented. The treatment of positive pregnant women, who were not penicillin sensitive, was benzathine penicillin G, 2.4 million units intramuscularly immediately as a single dose in accordance with Burkina Faso Ministry of Health (MOH) guidelines. In the national guideline the alternative treatment to penicillin is erythromycin 500mg 1 tablet 4 times per day during 14 days in addition to Polyvidone – iodine for ulceration.

Discussions about the eligibility criteria for women who would receive the test, the design of the intervention and the program manual were held during the workshop. The booklet-format manual was elaborated by the research team and included general information on syphilis, how to use of rapid diagnostic test (RDT) for syphilis, the purpose of the study, criteria for eligible women, obtaining consent, instructions for filling registers, and quality control.

There was no additional health educational campaign beside the routine health education organized by the facility or nongovernmental organization in the district.

### Conceptual framework

We adapted and applied the Normalization Process Model (NPM) proposed by May et al. [22]. NPM focuses on the workability and integration of the components of an intervention in everyday health care practice and identifies conditions that need to be addressed in order to facilitate health intervention integration into routine care. It assists process evaluation in two ways. First, it allows identification and description of factors that have been shown to be important in promoting or inhibiting the implementation of interventions. Second, it provides a basis for assessing the likelihood that an intervention will become routinely incorporated in practice. We sought to identify barriers and facilitators for the introduction of a rapid test for syphilis screening among pregnant women and to understand the conditions under which such an intervention could become routinely incorporated in the antenatal care package.

### Data collection

Data were collected through qualitative and quantitative methods.

Registers were given to health workers as record tools. In that register socio-demographic characteristics of women, test result and medication given were recorded. Interviews were conducted among health workers (*n* = 12) who used the point of care test for syphilis and district managers (*n* = 2). We sought to explore health workers’ opinions on organizational and managerial issues and their experience with point-of-care test for syphilis. Non-participant observations were conducted during ANC visits. The aim was to observe how ANC was offered and how RDT was used. A researcher sat in the consultation room during ANC from the time a woman entered until the end of her consultation. Observations (*n* = 14 ANC 1 observed) included listening and asking questions to health worker as they arose out of observations in order to gain insight into what was observed.

### Data analysis

Interviews were transcribed into a text program and then uploaded on Nvivo software. An analytical grid of key themes was developed based on the interview guide, the objectives of the research and familiarization with the first few transcripts. We explored the likelihood that point of care test for syphilis will become routinely incorporated in practice through four factors identified by the Normalization Process Model. These factors are interactional workability, relational integration, skill-set workability, and contextual integration [22].

Descriptive analysis was performed with quantitative data from registers.

### Ethical considerations

This research was approved by the University of Western Cape, the National Ethics Committee for Health in Burkina Faso and the Ethics Review Committee of the WHO. In addition, the study team obtained permission to conduct the intervention and research from the District authorities. Written consent was obtained from pregnant women who received the test by a health worker. They gave a witnessed signature or thumb-printed approval to participate. Moreover, we obtained consent from pregnant women and health workers during observations. The researcher explained to each woman the objective of the study in the waiting room. The consent process included an explanation of the study, its objectives, potential benefits and risks, confidentiality, and the voluntary nature of participation including the right to withdraw at any time.

## Results

### Implementing and evaluating the intervention

#### Organizational issues

Seven health facilities participated in the intervention. Data were compiled from 6 facilities because one facility did not complete the record tools. The intervention was implemented in the maternity unit over 4 months in 4 facilities, 2 months and 1 month in 2 facilities. For these latter facilities, stock outs and lack of communication among staff were the main difficulties. The intervention was integrated into the existing package of antenatal care services at primary healthcare level of the district, using the available staff. Syphilis test was performed during ANC. In many cases, it was delivered at the same time as the HIV test. The health worker pricked a woman’s finger once for a blood sample for both syphilis and HIV tests. In a few cases health workers pricked twice. There was no change in the organization of the health facilities and patient flow. The main complaint was the filling of the register for syphilis test. Health workers reported that there were many procedures and items to complete. In addition, some health workers highlighted an increase in the length of antenatal consultation. From our observations the difference in time between before and after the intervention was approximately 3 min.

#### Training

A one day on-site practical training was organized in each selected facility. The training was held in the maternity unit with all the staff available the day of the training. It covered the use of an onsite rapid syphilis test, filling of register book, case management of test-positive syphilis cases and quality control. After training, a simple manual, in French, was given to ensure standardization in screening and treatment for all trainees. In total 18 health workers such as midwife, auxiliary midwife and nurse received the training across 7 facilities. A pharmacist working for IRSS laboratory and the lead researcher travelled to selected facilities to provide the training.

All health staff found the training interesting and useful mainly because it was a refreshment of their knowledge. They also appreciated the onsite session because this avoided absence of staff and benefited all staff simultaneously, although due to turn over of health workers, some did not receive training. Nonetheless, some of the staff (03) who did not receive training performed the test.

#### Provision of supplies and drugs

The initial provision of rapid tests and ancillary supplies occurred on the same day, after the training. Thereafter, supplies were ordered by the research team and distributed to facilities by the health system’s district management team. The ancillary supplies comprised cotton wool, lancets, gloves, capillary tubes with EDTA, buffer and alcohol.

In addition to the kits provided by the national committee for HIV and STI control, the research team provided kits that comprised benzathine penicillin, erythromycin and polyvidone- iodine. Each facility received 7 kits.

#### Case management and partner notification

All eligible women were offered the syphilis test in the consultation room. Screening was free of charge for all women. Women at their first antenatal visit during the intervention period were eligible for the test. The decision to offer treatment was based on the point of care test result. Kits were available for treatment. All women diagnosed with syphilis were treated free of charge.

Women with syphilis were advised by the health worker to inform their partner or to invite them for a visit at the health facility without any letter or card of invitation. All health workers declared that partners did not respond to the invitation.

#### Quality control

All the seven facilities were involved in the quality control tests which were performed by the laboratory of the regional hospital located in Kaya. Supplies such as syringes, cryotubes were provided to health facilities and reagents (RPR and TPHA) and other supplies were provided to the laboratory.

Every 20^th^ negative and all positive rapid tests were to be followed immediately by venipuncture with blood samples sent to the laboratory. RPR and TPHA were the gold standard for the diagnosis of syphilis. Transport of blood samples from the health facility to the laboratory was conducted by health worker. Transport expenditure was reimbursed.

In sum, only 4 facilities sent at least one sample to the laboratory of the regional hospital for control. In total 12 samples were sent. Among these samples 8 were positive and 4 negative. According to the laboratory record, 10 samples were appropriate for analyzing, the others were hemolyzed. There was no discordance between the rapid test results and TPHA one. The small number of samples sent for validation is due to misunderstanding of health workers about the 20^th^ negative sample (some health workers misinterpreted the guidance as the 20th patient) and the system of reimbursement of transport costs.

#### Supervision and monitoring

For supervision, each health facility was visited once a month. A detailed checklist was elaborated for supervision. A separate register book for screened women was kept in each health facility. Records included the essential information as serial registration number, patient name, sociodemographic information, result of test, reasons for partner notification or non-notification. Supervision was perceived as important but not sufficient in some cases where staff did not follow the guidelines of the intervention despite many reminders. Supervision was also useful for supplies provision such as tests and treatment kits. In addition to visits to facilities, the team had monthly phone contact with the head of the maternity unit, with additional calls if there was any difficulty such as filling in the register, criteria for quality control or other issues. During supervisory visits, difficulties were discussed and patient register books were checked.

#### Effectiveness

Of 812 pregnant women who came for their first visit 39% were screened during the period of the intervention (Table 1). Rural facilities had higher coverage (66.8%) than the urban ones (25.6%). Among these screened women, 5.7% were positive.

**Table 1:** Percentage of pregnant women screened through the intervention

	Expected	Screened	Percent
CSPS rural 1	119	97	81.5
CSPS rural 2	63	40	63.5
CSPS urban 1	76	40	52.6
CSPS rural 3	83	40	48.2
CSPS urban 2	124	39	31.5
CSPS urban 3	347	61	17.6
Total	812	317	39.0

#### Barriers and facilitators

Interviews with health workers allowed collecting information on barriers and facilitators to introducing this new procedure into routine care.

#### Barriers

The perceived barriers were lack of training of all staff, workload, and lack of motivation.

Regarding lack of training for all staff as a barrier to introducing syphilis test, an auxiliary midwife in urban facility declared that “*You have to train everybody, because we work together. It could happen that the person who received the training is absent. You are here and would like to work but you have to call the person to ask what to do. If you are with a pregnant woman, she will wonder if you know your work.”* The lack of training prevents health workers from performing new tasks. Moreover health workers without training will not be able to address questions raised by women during consultation.

Some health workers found that the increase in workload is a barrier as illustrated by a midwife in charge of maternity unit in urban health facility “*People find that the workload is high with PMTCT here, malaria RDT there, with the filling of registers mainly with addition of PBF (Performance Based Financing), people find that the workload is too high.”* In fact the main complaint was the filling of the register for syphilis test. Staff felt that they already have many registers (4) and there was no need to add another. In addition, some health workers highlighted an increase in the length of antenatal consultation.

Another reported barrier was frequent stock-outs of consumables, which is also a source of demotivation. Consumables such as gloves and chase buffer were frequently claimed by health workers to be unavailable. The availability of chase buffer was crucial for the continuity of the activity in the health facility. Gloves were also important but when there was no free stock health workers said that pregnant women are required to buy them at the pharmaceutical store of the facility. It becomes a barrier when woman does not have enough money to purchase gloves. Stock-outs of consumables were a common reason that health workers offered for not screening women.

Lack of supervision influences motivation of health workers to perform task. One nurse in charge of a rural facility declared: “*if we start something without monitoring, at some point, staff will wonder if people who initiate the activity are interested in it. Thus there will be a demotivation for that activity*”.

#### Facilitators

All health workers found that offering the rapid test was a satisfying experience for them. Among facilitators, health workers cited political environment, ease of use and acceptability to pregnant women.

In terms of political environment, syphilis screening is officially part of the package of ANC in the guideline for ANC in Burkina Faso. In addition, there are new policies or reforms such as performance based financing (PBF) in which offering the full package of ANC is important. This is a good opportunity for implementation of syphilis screening as a routine activity as illustrated by this auxiliary midwife in rural facility “*With PBF, syphilis is part of [indicators that count in the evaluation]. If we get it (syphilis test), it will help us, we will gain points.*”

All health workers agreed that the rapid test was easy to use due to the similarity with the HIV testing. The comment of a midwife in an urban facility illustrated the ease of use: “*We did not face difficulties in performing testing as we did it for PMTCT, it is the same thing, we tick and collect blood, so it is the same thing. There was no difficulty because we received training for PMTCT.*” In addition, some health workers declared that they received support from other colleagues who received training.

Acceptability to women is important to the success of an activity. There was a good acceptability of the test and the health workers recorded few refusals from women. Health workers were reported that women agree to testing because they would like to know more about their health status and protect their fetus. Moreover a nurse in charge of rural facility argued that “*HIV screening is already performed, women know that there are different tests at CSPS level. Thus, asking a woman to do syphilis test will not be a problem. They are used to the different tests we deliver here, mainly HIV, it is a routine.*” In other word, pregnant women have got used to point of care tests like urine, HIV or malaria test and this is a favorable factor for introducing a new test in the package of ANC.

## Discussion

The study demonstrated that it is operationally feasible and acceptable to staff to integrate a point of care test including rapid on-site syphilis testing and prompt treatment of syphilis cases at primary healthcare level in Kaya health district, Burkina Faso. It showed that rapid diagnostic test allowed to have better detection of syphilis during ANC compared to the symptom based system. It also highlighted implementation challenges and barriers to routine implementation that need to be addressed prior to rolling out the intervention.

In developing countries, one of the gaps in syphilis control in pregnant women is the access to syphilis testing at peripheral facilities. To address this challenge, a pilot model with a point of care test was designed and tested in Burkina Faso. This pilot model included different components including onsite training, quality control and supervision. Decentralized models integrating a point of care for syphilis in ANC were designed and experimented in Low and Middle Income Countries (LMIC). These models were mostly implemented in Asia [23, 24], Latina America [25, 26] and eastern and southern Africa [27–31]. Few were implemented in West Africa [32]. Each model was adapted to the contextual health system and was feasible. This underlines the importance of contextual specificities in the implementation of health interventions and pilot studies.

In terms of training, onsite training was chosen instead of the usual off-site approaches. Off –site training has known limitations like absence of staff during training, small numbers of beneficiaries and that often it is the head of the facility and not front line workers who are targeted. Onsite training was chosen to limit absence of the staff from their facility and to offer a chance for all staff at maternity units to be trained to avoid the situation, common with off-site training as seen with HIV services, that staff who did not receive the training could use this to justify the non use of the rapid diagnostic test [33]. Nonetheless some health workers complained about not having received training. Most of these were absent during the training session or had been newly appointed at the facility. It is important, in case of scale –up of the intervention, to plan refresher training and to explicitly include mechanisms for trained staff to train new or untrained staff. Those who performed the test without training reported that it was due to the similarity with HIV testing.

Case management of positive pregnant women was not reported to be a problem for health workers. The main challenge was partner notification. In most of the cases, the patients’ husbands did not come to the facility as reported in many studies [34]. This is one of the limits of patient driven notification and may be particularly challenging in patriarchal societies where polygamy is common, such as Burkina Faso. Further research might study constraints to partner notification in such contexts and develop a workable and acceptable partner notification model [35].

With respect to the definition of integration of services understood as “the organization and management of health services so that people get the care they need, when they need it, in ways that are user friendly*,..”* [36], integration of syphilis services was observed at ANC units in this study as a “one stop shop”. Rapid syphilis testing and treatment were delivered during ANC within the examination room. At the staff organization level, there was no specific additional mechanism put in place. Despite the high reported acceptability of the test by health workers, there were 61% of missed opportunities. Missed opportunities could be explained by insufficiently clear instructions, lack of financial incentives in a context where some health workers have come to expect financial incentives for adding new tasks [37, 38], increase in duration of consultation and high number of patients or workload as found by Zongo et al. [39] in a study on utilization of RDT for malaria conducted in primary health care facilities in Burkina Faso.

The quality control was a challenging component because in the routine system the relationship between health workers at primary health facilities and lab personnel is limited to referral for laboratory tests that are not available at primary health facility. These cadres do not really collaborate except during HIV serosurveillance surveys where health workers at health facility send blood samples. The laboratory does not conduct any routine supervision to validate rapid diagnostic tests that are delivered at the peripheral level. For the implementation of the quality control during our study, health workers received reimbursement for their transport depending on the distance. In two facilities where there were delays in reimbursement, the activity was stopped. This finding underlines that the issue of health workers’ motivation in quality control needs to be properly addressed. In addition there is a need to implement a rigorous quality control, with regular supervision from laboratory personnel, in order to improve utilization of rapid diagnostic tests as a whole. The use of a rapid diagnostic test at level like primary health facility by non-lab personnel is a form of task shifting and needs supervision from lab personnel.

Most studies that documented syphilis screening programs did not pay particular attention to the workability and integration of the syphilis testing in ANC services. Our research identified facilitators and barriers that need to be addressed in order to facilitate integration of rapid test for syphilis into routine ANC services.

At the health worker level, respondents agreed that the rapid test for syphilis is easy to use because it is similar to HIV testing and is therefore compatible with their daily activities in ANC. The barriers to the full integration of the test are availability of consumables, filling of forms and lack of supervision. These factors - especially stock-outs of consumables - influence health workers’ motivation to deliver the service. Due to lack of supplies, staff frustration was underlined in HIV services at peripheral facilities in Burkina Faso [33]. Regular supply and supervision could help in maintaining motivation to deliver testing.

At the patient level, acceptability of testing is reported to be high. According to health workers, this high acceptability is due to their knowledge of existing testing such as HIV or malaria. The few refusals come from women who need prior agreement from their husband. However, the high acceptability hides constraints that oblige women to accept any service such as tests from health workers. From our observational survey of ANC, the interaction between health workers and pregnant women was unidirectional as Pembe et al. found [40]. The health worker talks and the pregnant woman nods or answers to questions. In such a frame, a pregnant woman does not have an opportunity to refuse any offer from health workers. This situation is corroborated by findings from Malawi where rural Malawians perceive routine testing for HIV at antenatal clinics as compulsory to receive antenatal care [41]. Moreover they found that constraining choice is likely when clients are women, rural and relatively uneducated compared to health personnel, as is the case in our study site. Although the situation helps staff in gaining high rate of acceptability of HIV testing and other services, the rights of clients may not be not fully respected.

Through the lens of the four factors of the Normalization Process Model (NPM), assessment of the likelihood that point of care test for syphilis will become routinely incorporated in practice was explored. These factors are interactional workability, relational integration, skill-set workability, and contextual integration.

In terms of interactional workability, the point-of care test for syphilis is both an opportunity to address a service gap and an opportunity to improve the standard of antenatal care. Health workers were aware that the current antenatal services did not provide optimal access to syphilis testing. This awareness could strengthen the willingness of health workers to consider its implementation. So, there is congruence with operational needs (difficult access to syphilis test) and antenatal care practice.

Regarding relational integration, the dimension of confidence was explored. Health workers are confident in the reliability of the test and the utility of the test. Health workers did not show any reticence regarding the reliability of the results. The results from quality control reinforce the confidence in those results. Their perceptions of the reliability could be influenced by similarity with HIV test. From our knowledge, health workers had a good perception of the reliability of HIV except in some cases of indeterminate results. While reliability could be a promoting factor, the utility of the syphilis test could be also considered as such. However this could be threatened by the low prevalence. Where the prevalence is low the utility for routine test could be questioned and reduce the willingness of health workers to systematically test all the patients.

Skill-set workability could be considered as a promoting factor in relation to the capacities of the health workers to perform the test and there being no change required in the division of labour. The challenge posed by the intervention was the increase in workload due to filling the register and the increased perceived duration of consultation. The existing routine ANC register and ANC card do not allow for filling in information on syphilis testing. This would need to be addressed as filling in separate syphilis registers is a significant disincentive. The perceived extension of ANC consultation time could be addressed by sharing the results of observation survey (only 3 min additional time) and drawing attention to possible improvement of ANC service quality. Supervision of the work by both district managers and laboratory personnel could also mitigate the effects of the challenge.

Contextual integration is favorable at international and national levels. At the international level, the global initiative for the elimination of congenital syphilis, launched by WHO [3], emphasized screening and treatment of pregnant women and their partners as a key component. At national level, policy and management of maternal care guidelines emphasize the importance of diagnosing and treating pregnant women for reproductive tract infections (RTIs). Moreover implementation of performance based financing with indicators concerning antenatal syphilis screening is a promoting factor. It could be argued that these initiatives provided a receptive international and local context for introducing point of care testing for syphilis in ANC services. Integration of point of care tests using available staff could contribute towards embedding this into routine practice. The main challenge was the provision of supplies. Supply chains in Burkina Faso are known to have difficulties that often lead to stock out of consumables.

In summary the study identified a number of facilitators to potential normalization including congruence with professional practice, confidence in the reliability and utility, capacities for performing the test and no change at organizational level.

One of the limits of the study is that there was no baseline data on the percentage of women who had their syphilis test at the laboratory. Available data do not allow linking women and health facilities. Thus comparison between before and after coverage was not possible. But we assumed that the coverage was increased as found in other studies [42]. Another limitation is that we were not able to show impact as our main focus was feasibility.

## Conclusion

In conclusion, point of care test for syphilis could be integrated in ANC at primary health facility in Burkina Faso. Nonetheless, barriers need to be addressed before scaling up. These barriers relate to staff motivation, which is negatively influenced by practical considerations such as stock-outs and the chore of filling in registers, inadequate supervision, and by some aspects perceived as unfair regarding training and incentives. Through the lens of NPM, point-of care test for syphilis is likely to be acceptable by health workers as routine service and incorporated as a normal practice, and may strengthen the quality of overall ANC services.

## Abbreviations

ANC: Antenatal Care
CSPS: Centre de santé et de promotion social
RDT: Rapid Diagnostic Test
